# Harnessing Treg Homeostasis to Optimize Posttransplant Immunity: Current Concepts and Future Perspectives

**DOI:** 10.3389/fimmu.2021.713358

**Published:** 2021-08-30

**Authors:** Shuntaro Ikegawa, Ken-ichi Matsuoka

**Affiliations:** ^1^Department of Hematology and Oncology, Okayama University, Okayama, Japan; ^2^Department of Medical Oncology, Dana-Farber Cancer Institute and Harvard Medical School, Boston, MA, United States

**Keywords:** regulatory T cell, graft-*versus*-host disease, interleukin 2, immune checkpoint inhibitor, post-transplant cyclophosphamide

## Abstract

CD4^+^CD25^+^Foxp3^+^ regulatory T cells (Tregs) are functionally distinct subsets of mature T cells with broad suppressive activity and have been shown to play an important role in the establishment of immune tolerance after allogeneic hematopoietic stem cell transplantation (HSCT). Tregs exhibit an activated phenotype from the stage of emigration from the thymus and maintain continuous proliferation in the periphery. The distinctive feature in homeostasis enables Tregs to respond sensitively to small environmental changes and exert necessary and sufficient immune suppression; however, on the other hand, it also predisposes Tregs to be susceptible to apoptosis in the inflammatory condition post-transplant. Our studies have attempted to define the intrinsic and extrinsic factors affecting Treg homeostasis from the acute to chronic phases after allogeneic HSCT. We have found that altered cytokine environment in the prolonged post-HSCT lymphopenia or peri-transplant use of immune checkpoint inhibitors could hamper Treg reconstitution, leading to refractory graft-*versus*-host disease. Using murine models and clinical trials, we have also demonstrated that proper intervention with low-dose interleukin-2 or post-transplant cyclophosphamide could restore Treg homeostasis and further amplify the suppressive function after HSCT. The purpose of this review is to reconsider the distinctive characteristics of post-transplant Treg homeostasis and discuss how to harness Treg homeostasis to optimize posttransplant immunity for developing a safe and efficient therapeutic strategy.

## Introduction

Allogeneic hematopoietic stem cell transplantation (HSCT) is a curative treatment strategy for patients with various hematological disorders (1). However, graft-*versus*-host disease (GVHD) remains a cause of significant morbidity and mortality following HSCT (2, 3). The pathophysiology of acute GVHD is the response of donor-derived effector T cells to recipient tissues, including the skin, gut, and liver. During the early phase after HSCT, these immune reactions also evoke dysregulated reconstitution of T and B cell subsets, leading to the basic pathogenesis of the development of chronic GVHD (4). Over the last decade, there has been increasing evidence showing that regulatory T cells (Tregs) play a critical role in the regulation of acute and chronic GVHD, and the robust reconstitution of Tregs is needed to reestablish a well-balanced immune system that can maintain appropriate levels of peripheral tolerance.

Tregs are a subpopulation of CD4^+^ T cells that specifically retain their immunosuppressive functions. Tregs were initially identified as CD4^+^CD25^+^ T cells (5), and subsequent studies have shown that Forkhead box P3 (Foxp3) is a master transcription factor for this subset (6). Mice lacking Foxp3 expression were seen to develop lethal autoimmune diseases (7), and humans with a mutation in the *Foxp3* gene are known to develop immune dysregulation, polyendocrinopathy, enteropathy, and X-linked syndrome, which is a severe multiorgan autoimmune disease (8). Tregs comprise 5–10% of peripheral blood CD4^+^ T cells in healthy individuals and play an essential role in maintaining peripheral self-tolerance and preventing autoimmune diseases (9, 10). In the context of allogeneic HSCT, CD4^+^CD25^+^ cells were initially found to be indispensable for immune tolerance against alloantigens using an *in vitro* mixed lymphocyte reaction assay (11). Later, using murine GVHD models, depletion of CD25^+^ cells from the donor inoculum exacerbated the severity of GVHD, and co-infusion of *ex vivo* cultured CD4^+^CD25^+^ cells resulted in significant inhibition of rapidly lethal GVHD *in vivo* (12). In addition to murine studies, the analyses of clinical samples from patients after HSCT demonstrated that the expression of the *Foxp3* gene in peripheral blood mononuclear cells was negatively correlated with the incidence and severity of clinical acute and chronic GVHD (13, 14). High Treg contents in the donor graft were associated with the low incidence of acute GVHD (15). Further, prospective monitoring of T cell reconstitution demonstrated that the unbalanced recovery of Tregs and effector T cells contributed to the development of chronic GVHD (16, 17). Notably, studies have shown that Tregs do not abrogate the cytotoxic activity of effector T cells against leukemia cells, both *in vitro* and *in vivo*, suggesting that Tregs could separate GVHD from graft-*versus*-tumor (GVT) activity mediated by donor-derived effector T cells (18). However, in a human clinical setting, CD25^+^ T cell-depleted donor lymphocyte infusion (DLI) showed a better disease control without increasing the incidence of acute GVHD than unmodified DLI in contemporaneous patients, indicating the possibility of Treg suppressive function against the GVT effect (19).

**Figure 1:**
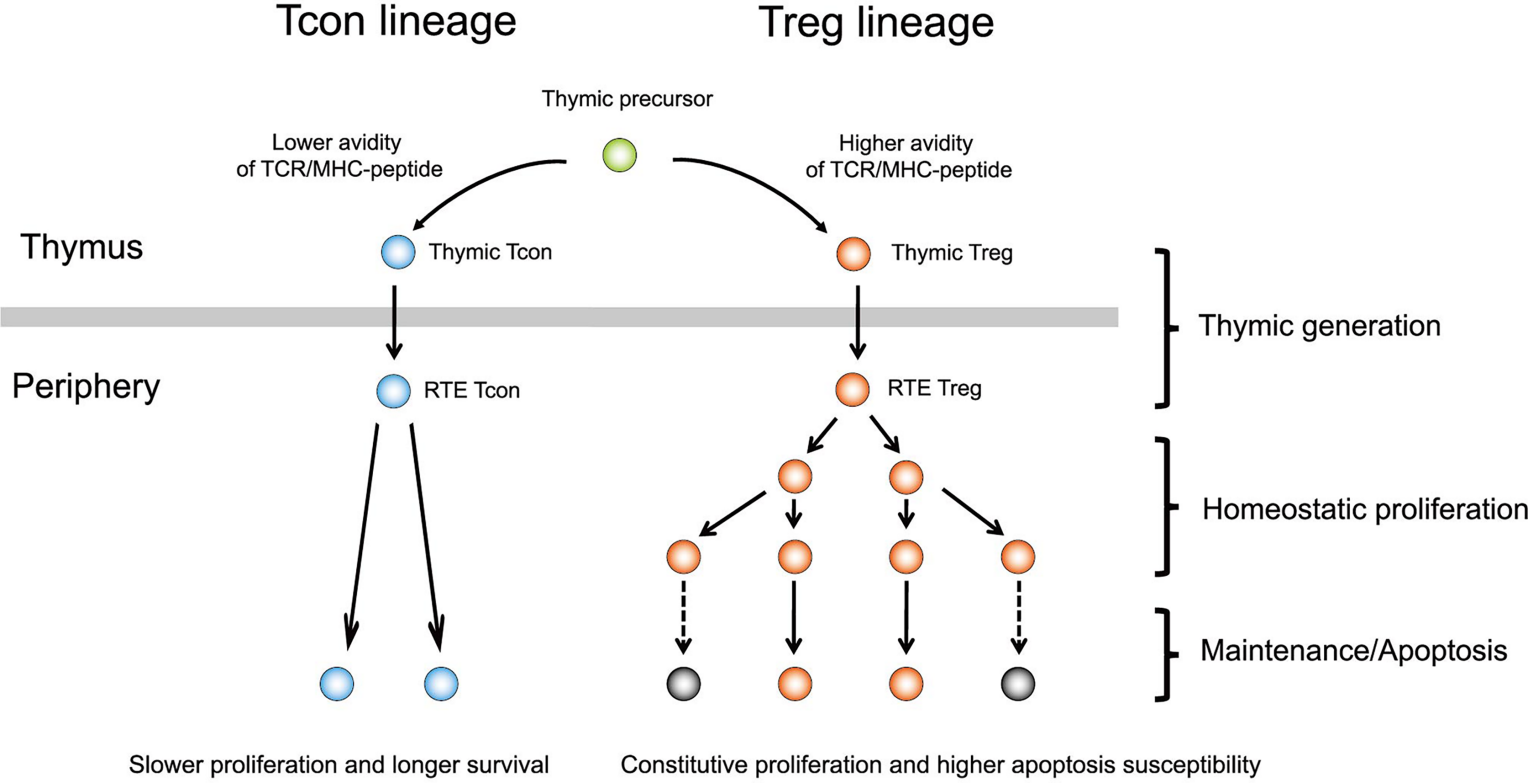
Development and maintenance of regulatory T cells. Tregs are positively selected from a population with a higher avidity of TCR/MHC-peptide in the thymus and emigrate to the periphery. Tregs already exhibit an activated phenotype at emigration to the periphery and undergo rapid homeostatic proliferation. Constitutive proliferation predisposes Tregs to apoptosis. In contrast, Tcons generally undergo slower proliferation and, thus, stable homeostasis with lower apoptosis. Tcon, conventional T cell; Treg, regulatory T cell; RTE, recent thymic emigrant; TCR, T-cell receptor; MHC, major histocompatibility complex.

**Figure 2:**
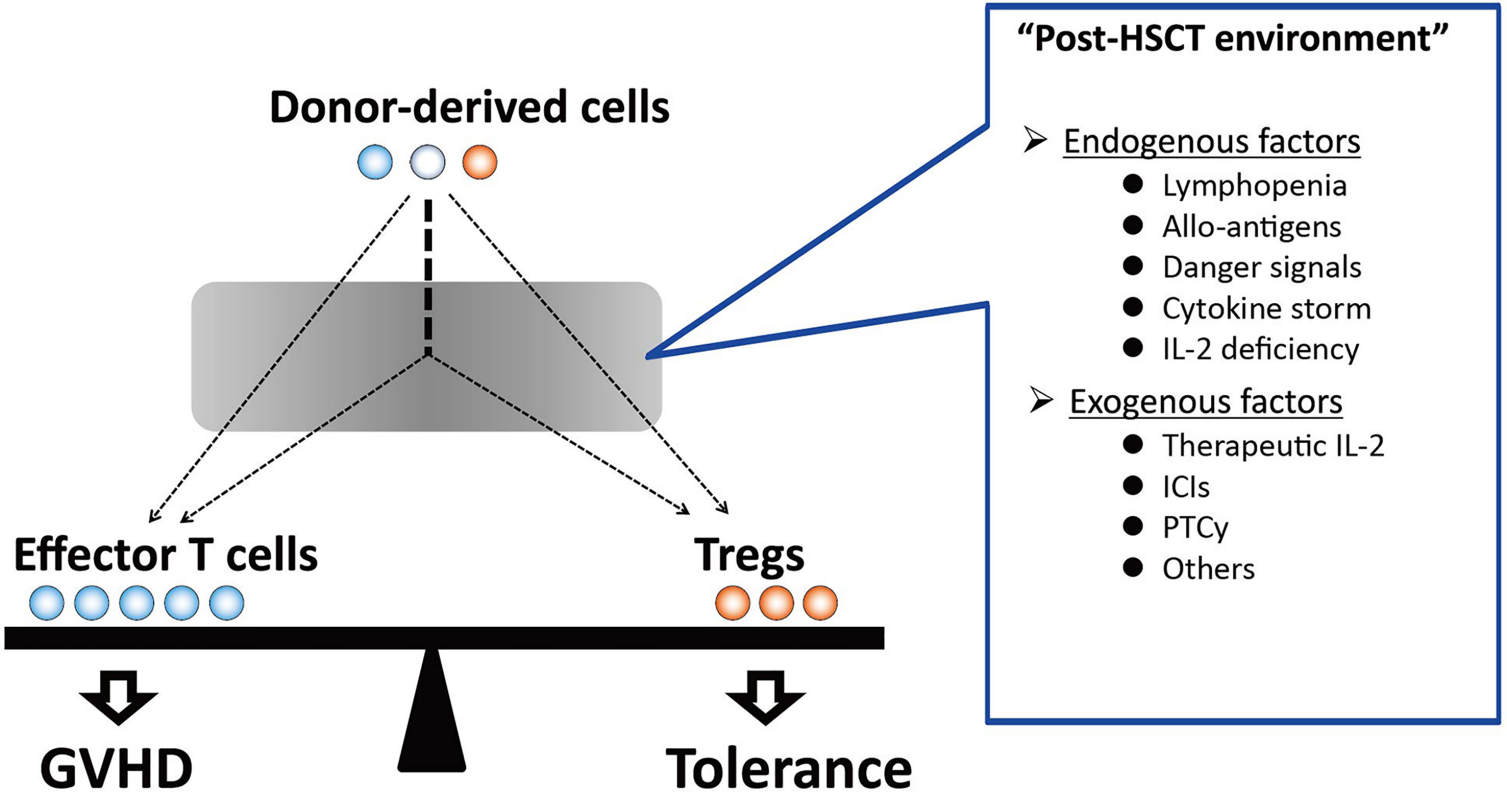
The balance between effector T cells and Tregs in the distinct post-HSCT environment. After HSCT, donor-derived T cells are exposed to a variety of endogenous factors, including lymphopenia, alloantigens, danger signals, and cytokine storm, which affect the reconstitution of donor-derived immune cells. In addition, therapeutic interventions that have immunomodulating effects during the peri-transplant period also affect donor-derived T cell homeostasis. Unbalanced immune reconstitution may provide a basis for the pathogenesis of GVHD. In contrast, favorable Treg recovery in the post-transplant environment results in the induction of immune tolerance. GVHD, graft-*versus*-host disease; Treg, regulatory T cell; HSCT, hematopoietic stem cell transplantation; IL-2: interleukin 2, ICI, immune checkpoint inhibitor; PTCy, post-transplant cyclophosphamide.

In general, human T cells express CD25 and Foxp3 after antigen presentation and undergo cell activation. In contrast, Tregs highly express such activated markers during migration from the thymus and maintain continuous proliferation in the periphery (**Figure 1**). The distinctive feature in homeostasis enables Tregs to respond sensitively to small environmental changes and exert necessary and sufficient immune suppression. However, on the other hand, it also predisposes Tregs to be susceptible to apoptosis. In patients after allogeneic HSCT, Tregs are exposed to a profound inflammatory environment affected by various endogenous factors, including severe lymphopenia, allogeneic antigen stimulation, and altered cytokine milieu. In addition to such endogenous factors after HSCT, various therapeutic immune modulators have been increasingly used in the peri-transplant period in recent years (**Figure 2**). Our studies have elucidated that both endogenous and exogenous factors have major impacts, particularly on Treg homeostasis and clinical outcomes after allogeneic HSCT.

In this review, we outline the characteristics of posttransplant Treg homeostasis and discuss how to harness Treg homeostasis to optimize posttransplant immunity. In particular, we would attempt to define the intrinsic and extrinsic factors that affect Treg homeostasis from the acute to chronic phases after allogeneic HSCT.

## Characteristics of Treg Homeostasis After HSCT

### The Generation and Maintenance of Tregs

Tregs are positively selected from populations with a higher avidity of T cell receptor (TCR)/major histocompatibility complex-peptide in the thymus and emigrate to the periphery (**Figure 1**). Therefore, Tregs can be considered as a physiologically preactivated population and show distinctive homeostasis in the periphery (**Figure 1**). Tregs have a high turnover and fine sensitivity to a variety of signals from the environment to regulate the cell number, localization, and function required to efficiently react to delicate changes in the immune system (20). Peripheral Tregs exhibit a high basal proliferation rate compared with conventional T cells (Tcons) in both mice (21) and humans (22); however, the high proliferation rate of Tregs is counterbalanced by a high rate of apoptosis (16). Interleukin-2 (IL-2), which is mainly produced by effector T cells, plays a pivotal role in Treg homeostasis (20). Importantly, Tregs cannot produce IL-2 independently; therefore, homeostasis critically depends on the extrinsic cytokine environment of the cell.

### The Reconstitution of T Cell Subsets and Immune Tolerance

The reconstitution of T cell subsets after HSCT is a polymorphic and long-term process. In general, the initial phase of T cell reconstitution is primarily dependent on the peripheral expansion of mature T cells that are contained in the stem cell graft (23). This process is promoted by lymphopenia-induced signals and the stimulation of donor T cells by alloantigens (16). Hematopoietic stem cells (HSC) also differentiate through the thymus, and thymus-derived T cells are then exported to the periphery. However, thymus-dependent generation of donor T cells is generally delayed and incomplete in adult patients because of natural thymic involution and damage, especially in thymic epithelial cells, resulting from high-dose chemotherapy and irradiation administered as part of the conditioning regimen (24–28). Once naïve T cells are exported into the periphery from the thymus, they are subject to homeostatic signals that regulate the expansion and contraction of the T cell population to maintain the total number of T cell subsets at an appropriate level. Although the transition of peripheral T cells from mature T cells in the stem cell graft into thymic-generated naïve T cells is commonly observed in each T cell lineage after HSCT; each T cell subset is subject to distinct homeostatic controls in the periphery (29). Particularly, Treg homeostasis appears to be distinct from Tcon, and this may contribute to an imbalance between Treg and Tcon (**Figure 2**) (10, 22). We previously studied long-term Treg reconstitution after clinical HSCT and the impacts on the development of chronic GVHD (16). Our data indicated that thymic generation of Tregs was markedly impaired, while this subset maintained a significantly higher level of proliferation as compared to Tcons. Treg proliferation *in vivo* appears to be driven primarily by CD4 lymphopenia. Importantly, high levels of Treg proliferation are counterbalanced by its increased susceptibility to apoptosis. Altered Treg homeostasis in response to the homeostatic pressure under prolonged CD4 lymphopenia resulted in the selective peripheral depletion of Tregs and the subsequent development of chronic GVHD.

A recent study that analyzed CD4^+^Treg heterogeneity with a large panel of functional markers using mass cytometry by time of flight revealed that in addition to the numerical deficiency, reduced heterogeneity of CD4^+^Tregs was associated with the development of chronic GVHD (30). Thus, the thymic generation of *de novo* Tregs from donor HSC plays an essential role in long-term immune reconstitution. In addition to Treg heterogeneity, foxp3 stability (31) is also important for Treg survival and GVHD suppression (32–34). The post-transplant inflammatory environment may affect the genetic stability of Tregs and should be taken into consideration for the understandings of Treg homeostasis after HSCT.

In addition to donor-derived Tregs, host-derived Tregs appear to play a crucial role in immune tolerance, especially during the very early phase after HSCT. Since Tregs are relatively radioresistant (35), murine studies showed that host Tregs could survive for several weeks after HSCT and contribute to suppressing the alloimmune response in the experimental mice model in the low level of irradiation before HSCT (36).

### The Basic Framework of Treg Reconstitution After Clinical HSCT

**Figure 3:**
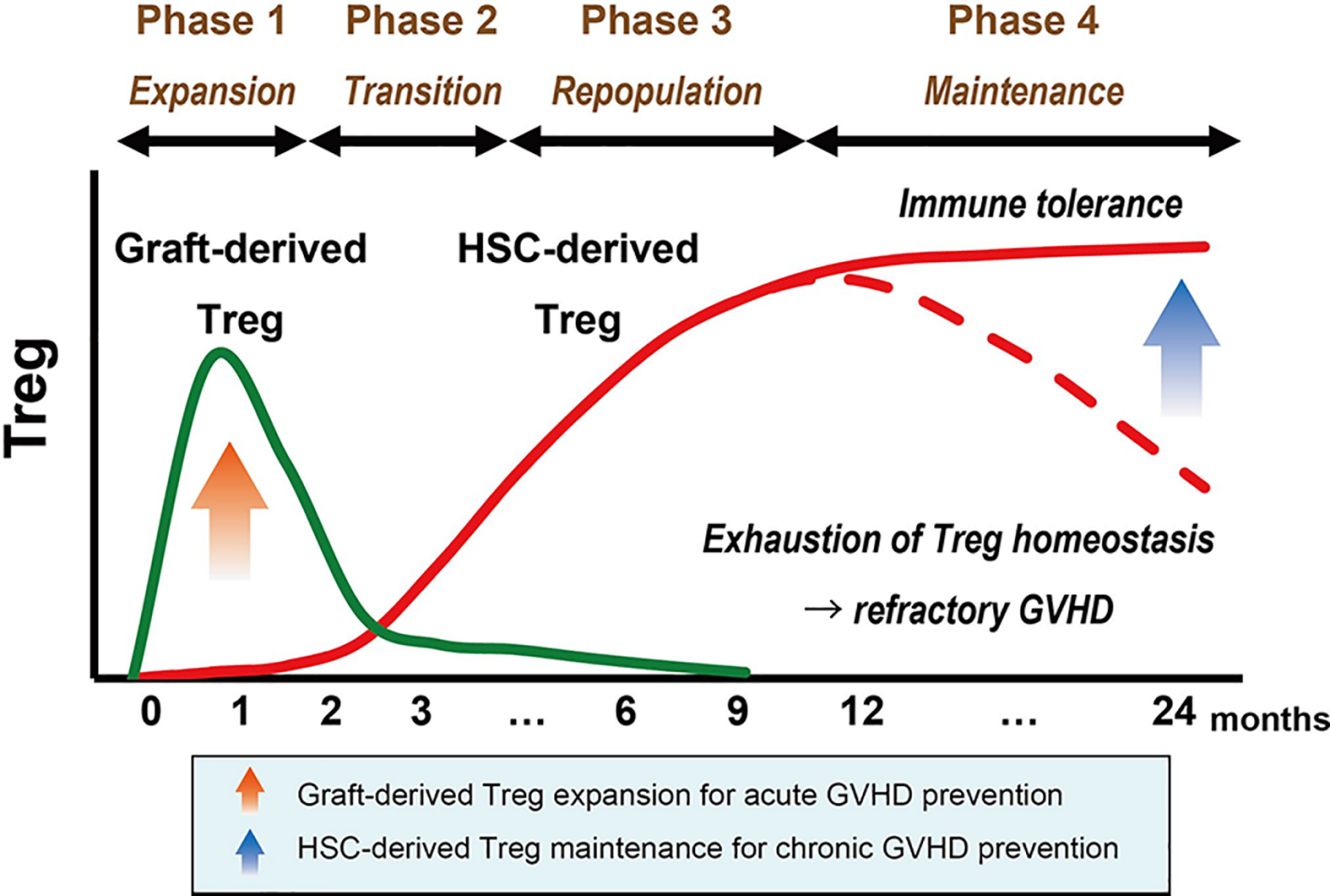
Basic framework of Treg reconstitution after HSCT. The basic framework of Treg recovery after HSCT consists of four phases. In the first month after HSCT, lymphopenia-driven peripheral proliferation of graft-derived Tregs (shown in green) induces the initial expansion of this subset, which is important for preventing acute GVHD (Phase 1). The total number of Tregs transiently decrease between the contraction of expanded graft-derived Tregs and the emergence of HSC-derived Tregs (shown in red) (Phase 2). Prolonged lymphopenia in the first year promotes the continuous aggressive proliferation of HSC-derived Tregs, which contributes to the recovery of total Treg cells and provides an essential basis for long-term immune tolerance (red solid line) (Phase 3). However, highly proliferative Tregs can fail to maintain homeostasis due to the high susceptibility to apoptosis, and exhaustion of the Treg population could be a trigger for refractory GVHD (red dotted line) (Phase 4). GVHD, graft-*versus*-host disease; Treg, regulatory T cell; HSC, hematopoietic stem cell.

Based on the above findings, we proposed a basic framework of Treg reconstitution after clinical HSCT (**Figure 3**) (37). It comprises four phases: Treg expansion, transition, repopulation, and maintenance. Although previous studies have suggested that host-type Tregs show a temporary expansion immediately after HSCT after non-myeloablative conditioning, the kinetics of host-Tregs after HSCT is left out from this schema to simply discuss the donor-Treg engrafting processes.

### Phase 1: Expansion of Graft-Derived Tregs

During the first month post-HSCT, donor stem-cell graft-derived mature Tregs promptly expand in response to lymphopenia and alloantigens. The expansion peaks around the first month after HSCT and appears to be contracted by activation-induced cell death (AICD). It remains unclear whether some part of graft-derived Tregs would survive as functional memory suppressor cells for a long time after HSCT (38).

### Phase 2: Transition of Peripheral Treg Source

The emergence of HSC-derived Tregs is generally delayed because of thymic dysfunction after HSCT; therefore, the total number of Tregs in the periphery decreases after the contraction of graft-derived Tregs (transitional phase). The decline in the number of Tregs in this phase may be associated with the late onset of acute GVHD or overlap syndrome.

### Phase 3: Repopulation and Relocation of Thymus-Generated Tregs

After a dip in the number of Tregs in Phase 2, it takes about a year for the number of circulating Tregs to increase and approach the normal range. It is much faster than the reconstitution of Tcon subsets, which often reach a normal range within over 2 years (16). Treg repopulation in this phase is mainly constituted by HSC-derived Tregs. Although thymic generation of Tregs is markedly impaired after HSCT, newly generated Tregs maintain higher levels of proliferation during this phase. Proliferation of Tregs is relatively aggressive as compared to other Tcons, resulting in high levels of the Treg/Tcon ratio and the Treg/CD8T ratio in this phase (16, 17). The recovery of thymus-generated Tregs is highly influenced by age-related natural thymic involution, thus Treg kinetics in Phase 3 should be carefully evaluated in the pediatric transplant cohorts.

### Phase 4: Maintenance of HSC-Derived Tregs

As only naïve Tregs are capable of aggressive proliferation, the stable emergence of naïve Tregs from the thymus is thought to be critical for the maintenance of Treg homeostasis. Tregs can repopulate by compensatory homeostatic proliferation in Phase 3, even in patients with severe thymic damage; however, high levels of Treg proliferation in prolonged lymphopenia are counterbalanced by increased susceptibility to apoptosis, 6–9 months after HSCT. Importantly, peripheral depletion based on abnormal homeostasis is observed only in Tregs, but not in other Tcons, resulting in the imbalance of lymphocyte subsets, which provides a fundamental basis for the development of chronic GVHD (16, 39, 40).

### Treg Migration After HSCT

Appropriate circulation and localization of Treg are crucial for systemic immune suppression. Luciferase-expressing Tregs enabled tracking the tissue migration and survival of Treg longitudinally in mice HSCT model. Transferred Tregs proliferated in secondary lymphoid organs and sequentially migrated and localized into peripheral tissue after allogeneic stimulation *in vivo* (41). The initial priming of Tregs occurring in the secondary lymph node required CD62L expression on Tregs to migrate into lymphoid organs and protect from GVHD lethality (42). The proinflammatory environment during the early period after HSCT is essential for early Treg expansion and migration to GVHD sites. These Tregs exhibited sufficient suppressive function for alloreactive effector T cell proliferation in lymphoid organ and peripheral tissue (41).

The above model of Treg reconstitution is based on the number of Tregs in peripheral blood after clinical transplantation (16, 17). Most clinical studies test Tregs in peripheral blood rather than lymph nodes or target peripheral tissues, while mouse studies often test Tregs in the spleen and lymph nodes. Therefore, interpretation of the results needs to be carefully considered. Tregs at each site after HSCT may have different properties and functions for the overall regulation of allogeneic immunity.

## Effects of Low-Dose IL-2 on Treg Homeostasis

### IL-2 and Treg

It was initially found that IL-2 stimulates naïve T cell proliferation and generates effector and memory T cells. Later, several reports have shown that the germline knockout of IL-2 (43, 44) or blocking of IL-2 (45) or CD25 (46) results in autoimmune diseases, suggesting that IL-2 plays an essential role in immune tolerance. After identifying Tregs, IL-2 is now known as a critical homeostatic cytokine for Treg function (47), Treg differentiation in the thymus, and Treg expansion in the periphery (48, 49). The IL-2 receptor (IL-2R) is expressed as an intermediate-affinity dimer (IL-2Rβ and IL-2Rγ) or high-affinity trimers, which include IL-2Rα (CD25). Tregs constitutively express high-affinity to IL-2 receptors, which enables Tregs to respond to a small amount of IL-2, which other T cell subsets do not respond to. Initially, low-dose IL-2 was used to prevent disease recurrence owing to the GVT effect from natural killer (NK) cells, which also express CD25 in a steady state (50). In this pioneering study, low-dose IL-2 (2–6 × 10^5^ IU/kg/day) was administered for up to 3 months, and only one patient developed acute GVHD. Although the effect of low-dose IL-2 on lymphocyte subsets could not be fully evaluated because Treg had not been defined at the time of the study, the follow-up study revealed that the use of low-dose IL-2 induced the increasing Tregs in patients (51). The results led to the subsequent project of low-dose IL-2 therapy which aimed to reconstruct immune tolerance by increasing Tregs in patients with chronic GVHD (52).

### Low-Dose IL-2 for Chronic GVHD Treatment

Based on the theory that Tregs preferentially respond to low-dose IL-2, we conducted a phase 1 trial of administering low-dose IL-2 therapy daily in patients with steroid-refractory chronic GVHD (SR-cGVHD). In this trial, a total of 29 patients were enrolled, and the maximum tolerated dose of IL-2 was 1 × 10^6^ IU/m^2^; none of the patients experienced progression of chronic GVHD or relapse of hematologic cancers. Of the evaluable 23 patients, 12 patients had a major response, and the number of CD4^+^Tregs was preferentially increased in all patients (53). A subsequent phase 2 trial evaluated the efficacy of daily IL-2 (1 × 10^6^ IU/m^2^) administration for 12 weeks in 35 adult SR-cGVHD patients. The treatment was well tolerated, with 20 of 33 evaluable patients demonstrating clinical response at multiple chronic GVHD sites. The phase 2 trial also showed a rapid increase in the absolute number of Tregs and a rapid increase in the Tregs/Tcons ratio (54).

To elucidate the biological mechanisms of low-dose IL-2, we examined the effects of daily IL-2 therapy on the homeostasis of CD4^+^ T cell subsets after transplantation (55). We first demonstrated that chronic GVHD is characterized by constitutive phosphorylation of Stat5 in Tcon, associated with elevated levels of IL-7 and IL-15 and relative functional deficiency of IL-2. This was promptly corrected after IL-2 therapy, which resulted in the selective increase of Stat5 phosphorylation in Tregs and a decrease in pStat5 in Tcons. This was associated with profound changes in Treg homeostasis, including increased proliferation, increased thymic export, and enhanced resistance to apoptosis (55). A single cell mass cytometry analysis confirmed that low-dose IL-2 preferentially activated p-STAT5 in Helios^+^ naïve Tregs (56), promoted an increase in the population, and enhanced the suppressive function. In addition to increasing the number of Tregs by low-dose IL-2, it may also be important to increase the T cell receptor (TCR) diversity of Tregs. Previous studies have already shown that the limited TCR diversity of Tregs may be associated with autoimmune diseases (57, 58) and GVHD in the experimental models (59). A recent study using samples from patients who received low-dose IL-2 demonstrated that the increased Treg diversity after IL-2 treatment was associated with the improvement of clinical symptoms of chronic GVHD (60). Taken together, low-dose IL-2 therapy restores Treg homeostasis and promotes the reestablishment of immune tolerance in patients with chronic GVHD.

To reconsider the optimum algorithm of IL-2 intervention for safe and efficient Treg expansion, we conducted a murine model study, and found that the daily administration might not be required and the intermittent administration within threshold could be sufficient for the maintenance of expanded Tregs after the initial intensive IL-2 intervention (61). Based on the idea, we have designed and started a multicenter phase I/IIa clinical trial of low-dose IL-2 for patients with SR-cGVHD (62). In the protocol, IL-2 treatment is composed of two sequential phases: the induction phase and the maintenance phase. In the induction phase, IL-2 is subcutaneously administrated once per day for 4 weeks. In the subsequent maintenance phase, IL-2 is subcutaneously administrated three times per week for following period up to 1 year. The study had been completed and showed the favorable results with the stable immunological and clinical effects (63). Data are now being analyzed for publication.

Further studies are needed to determine the optimal dosage, timing, duration, and combination of low-dose IL-2 therapy in patients with chronic GVHD. Recently, engineered Treg-selective human IL-2 was developed in humanized mice during xenogeneic GVHD (64), and Efavaleukin Alfa (AMG-592), a human IL-2 mutant designed to have greater Treg selectivity and longer half-life compared with recombinant IL-2, is now under a clinical trial for patients with SR-cGVHD (NCT03422627). Additionally, a phase 1 clinical trial focusing on the combination of donor-Treg infusion followed by low-dose IL-2 for SR-cGVHD is also in progress (NCT01937468).

## Effects of Immune-Checkpoint Inhibitors on Treg Homeostasis

Immune checkpoints play an important role in the activation of effector T cells and the regulation of cytotoxic activity. In addition to this, previous studies have shown that they play a pivotal role in the homeostasis and function of Tregs, which originally exhibit an activated phenotype.

PD-1 is an immune checkpoint receptor that attenuates T-cell activation by interacting with its ligands, PD-L1 and PD-L2 (65). However, the exact role of PD-1 in the immune suppressive function of Tregs remains unclear. PD-1-deficient Tregs improved the symptoms of autoimmune pancreatitis in a mouse model (66), while in human glioblastoma tissues, Tregs expressing a high level of PD-1 had an exhausted phenotype and reduced immunosuppressive function (67). Moreover, in patients with gastric cancer who developed hyper-progressive disease after PD-1 blockade, tumors possessed highly proliferative Tregs after treatment (68). These studies suggest that PD-1 may regulate the immunosuppressive function of Tregs. In contrast, in a mouse chronic infection model, PD-1 blockade attenuated immunosuppression by Tregs, mediated by direct interaction of PD-1 on Tregs with PD-L1 on CD8^+^ T cells (69). These discrepant results regarding the role of PD-1 on the suppressive function of Tregs might be explained by the differences in the class of Tregs (70).

Cytotoxic T-lymphocyte-associated protein 4 (CTLA-4) is an inhibitory checkpoint molecule. Tregs constitutively express a high level of CTLA-4, whereas Tcons express CTLA-4 only after activation (71). CTLA-4 competes with CD28 for CD80/CD86 ligands, resulting in the inhibition of co-stimulatory signals (72). CTLA-4 blockade was the first immune checkpoint inhibitor (ICI) approved for clinical use, and the mechanisms of the anti-CTLA-4 blockade were distinct from those of PD-1 blockade (73, 74). Regarding the effect of Tregs, several preclinical studies indicated that the anti-tumor effects of CTLA-4 blockade were dependent on the depletion of CTLA-4-expressing Tregs in the tumor microenvironment *via* antibody-dependent T cellular cytotoxicity, leading to an increase in the CD8^+^/Treg ratio (70). However, anti-CTLA-4 immunotherapy for patients with solid cancers did not deplete Tregs (75); therefore, further analyses to address the role of CTLA-4 in Tregs in various settings are warranted.

### The Effect of PD-1 on Treg Homeostasis During Low-Dose IL-2 Therapy

**Figure 4:**
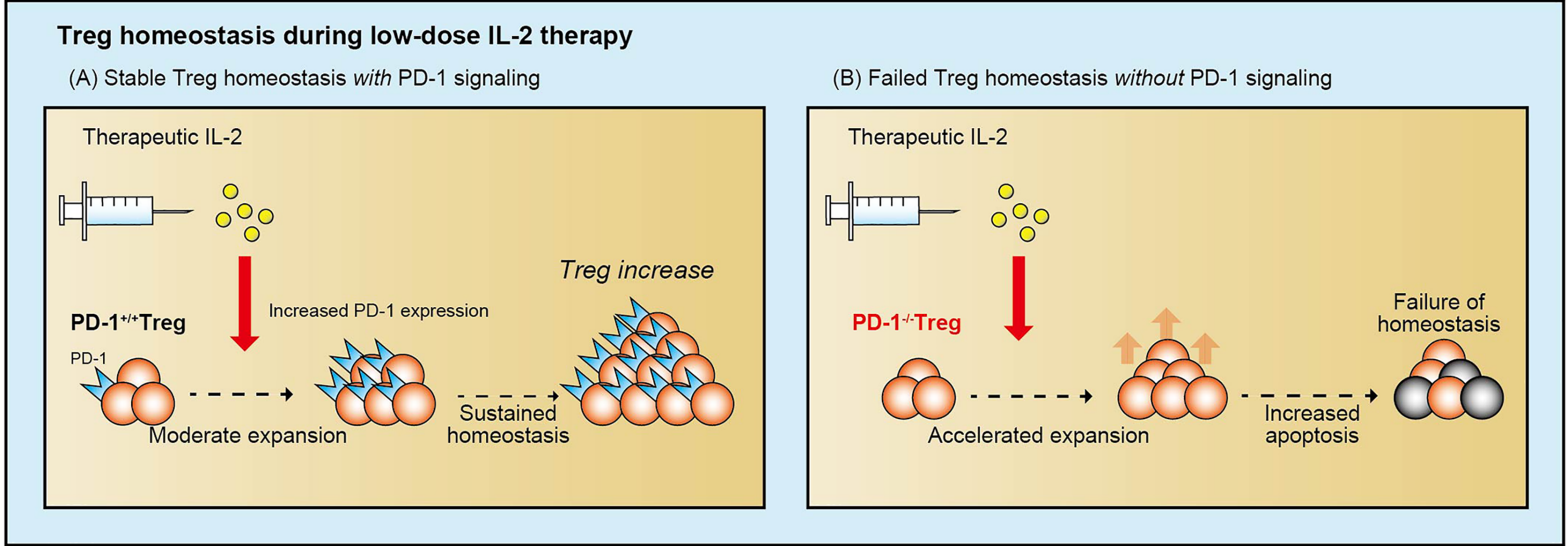
The role of PD-1 in Treg homeostasis during low-dose IL-2 therapy. Low-dose IL-2 therapy increases PD-1 expression on Tregs, and PD-1 preserves sustainable Treg homeostasis during low-dose IL-2 therapy, resulting in the preferential expansion of Tregs and the clinical improvement of chronic GVHD symptoms **(A)**. In contrast, without sufficient PD-1 signaling, low-dose IL-2 therapy accelerates Treg expansion soon after initiation, but Tregs are predisposed to the proapoptotic status and cannot maintain the expansion **(B)**. IL-2, interleukin 2; PD-1, programmed cell death 1; Treg, regulatory T cells.

Low-dose IL-2 therapy increased circulating Tregs and improved clinical symptoms of chronic GVHD; however, the mechanisms that regulate Treg homeostasis during IL-2 therapy have not been well studied. To elucidate these regulatory mechanisms, we examined the role of inhibitory coreceptors on Tregs during IL-2 therapy in a murine model and in patients with chronic GVHD (76). Murine studies demonstrated that low-dose IL-2 selectively increased Tregs and simultaneously enhanced the expression of PD-1, especially on CD44^+^CD62L^+^ central-memory Tregs, whereas the expression of other inhibitory molecules, including CTLA-4, LAG-3, and TIM-3 remained stable. PD-1-deficient Tregs showed rapid Stat5 phosphorylation and proliferation soon after IL-2 initiation; however, Tregs became proapoptotic with higher Fas and lower Bcl-2 expression. As a result, the positive impact of IL-2 on Tregs was completely abolished, and Treg levels returned to baseline despite continued IL-2 administration (**Figure 4**). We also examined circulating Tregs from patients with chronic GVHD who were receiving low-dose IL-2 and found that IL-2-induced Treg proliferation was promptly followed by increased PD-1 expression on central-memory Tregs. Notably, clinical improvement of GVHD was associated with increased levels of PD-1 on Tregs, suggesting that the PD-1 pathway supports Treg-mediated tolerance. These studies indicate that PD-1 is a critical homeostatic regulator of Tregs by modulating proliferation and apoptosis during IL-2 therapy.

### The Effect of PD-1 on Treg Homeostasis After HSCT

It has been observed that PD-1 blockade therapy is an effective strategy for hematological malignancies such as classical Hodgkin lymphoma (cHL) and it has made it possible to bridge chemotherapy-refractory patients to allogeneic HSCT (77–79). However, PD-1 blockade during the peri-transplant period theoretically enhances the donor effector T cell response, leading to an increased risk of severe GVHD. A pioneering murine model study demonstrated that PD-1 blockade potently enhanced T cell alloresponses both *in vitro* and *in vivo*, and the effect of PD-1/PD-L1 blockade was largely dependent on interferon (IFN) production (80). A subsequent study evaluating the role of PD-1 ligand for GVHD demonstrated that PD-1/PD-L1 blockade, but not PD-1/PD-L2 blockade, markedly accelerated GVHD lethality; suggesting an important differential role of host PD-L1 and PD-L2 in controlling GVHD (81). In the clinical setting, retrospective studies reported that pretransplant PD-1 blockade increased the risk of severe acute GVHD (82–84). Nieto et al. investigated clinical samples of patients treated with nivolumab before HSCT and demonstrated that nivolumab was detectable in the plasma for up to 56 days after HSCT was performed, and this residual nivolumab could bind to and block PD-1 expressed on donor effector T cells during this period. They also showed that pretransplant nivolumab resulted in a high frequency of IFN-γ-producing effector T cells, which might contribute to severe GVHD after transplantation (85).

**Figure 5:**
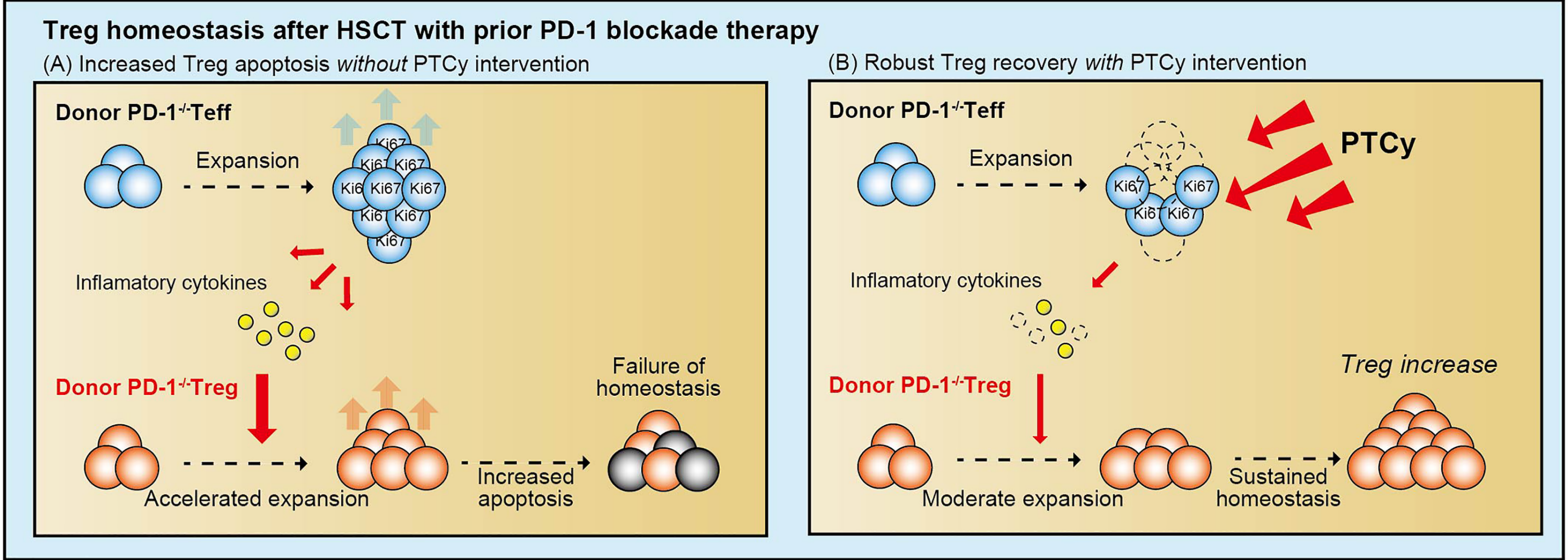
The role of PD-1 in Treg homeostasis after allogeneic HSCT. PD-1 deficient effector T cells aggressively proliferate after HSCT and produce large amounts of inflammatory cytokines. The highly inflammatory cytokine milieu accelerates the proliferation of PD-1 deficient Tregs, but they cannot maintain proliferation due to their high susceptibility to apoptosis. Unbalanced reconstitution of T-cell subsets results in severe GVHD **(A)**. In contrast, with PTCy intervention, alloreactive proliferative effector T cells are efficiently eliminated, contributing to reduced inflammatory cytokine production. It enables Tregs to restore the appropriate levels of proliferation and maintain homeostasis, which contributes to the prevention of severe GVHD **(B)**. PD-1, programmed cell death 1; Treg, regulatory T cell; Teff, effector T cell; PTCy, post-transplant cyclophosphamide.

Our group examined the effect of PD-1 blockade on Treg homeostasis after allogeneic HSCT in murine models and human clinical samples. First, we demonstrated that pre- and peri-transplant PD-1 blockade increased the severity of GVHD due to the unbalanced reconstitution of T cell subsets in recipient mice with PD-1 inhibition (86). PD-1^-/-^ effector T cells aggressively increased after HSCT, whereas PD-1^-/-^ Tregs could not maintain the expansion due to high susceptibility to apoptosis, leading to an unbalanced reconstitution of T cells, resulting in lethal GVHD. Interestingly, post-transplant cyclophosphamide (PTCy) restored the well-balanced reconstitution of T cell subsets and prevented tissue damage after HSCT from donor PD-1^-/-^ effector T cells. Based on fundamental data from a murine study, we examined clinical samples from patients who underwent HSCT. The clinical sample analyses revealed that PTCy promoted vigorous recovery of Tregs in recipients who underwent HLA-haploidentical transplantation following nivolumab therapy (87). These results suggest that PD-1 plays a crucial role in Treg homeostasis, especially in the early phase after HSCT. Moreover, PTCy might be an optimal GVHD prophylaxis when regulatory PD-1 signaling is functionally abolished by therapeutic intervention before HSCT (**Figure 5**).

In fact, recent clinical data have clearly indicated that PTCy was associated with a low incidence of severe GVHD in recipients with pre-transplant ICIs (88–91). Furthermore, Merryman et al. recently reported the results of an international retrospective study that included 209 relapsed or refractory cHL patients who underwent HSCT after PD-1 blockade (91). They demonstrated that PTCy-based haploidentical HSCT was associated with significant improvements in the progression-free and relapse-free survival of GVHD as compared with HLA-matched HSCT without PTCy. This suggests that pretransplant ICI and subsequent PTCy-based HSCT may provide better GVHD control along with better disease control (88–91). Further investigations on the impact of peri-transplant ICIs and PTCy on GVT activity are warranted.

### The Effect of PTCy on Treg Homeostasis After HSCT

PTCy is a novel GVHD prophylactic strategy for acute GVHD after HSCT from an HLA-haploidentical donor (92), and has been extended to an HLA-identical donor as a single agent GVHD prophylaxis (93–95). The mechanisms underlying the effect of PTCy involve impaired function of alloreactive T cells (96), preserving hematopoietic stem cells and Tregs by their high expression of aldehyde dehydrogenase (97). The indispensable role of donor-derived Tregs in preventing GVHD in PTCy was shown in a mouse model (98), which suggests that PTCy-based GVHD prophylaxis may preferentially promote the reconstitution of Tregs in a clinical setting. However, there are limited data on Treg reconstitution after HSCT with PTCy-based GVHD prophylaxis using human clinical samples (99–102). Our and other groups recently reported the reconstitution of lymphocyte subsets between HLA-haploidentical PTCy-based HSCT (PTCy-haplo) as compared to HLA-identical donor HSCT using conventional GVHD prophylaxis (101, 102). These studies demonstrated that the number of T cells was significantly lower in PTCy-haplo than that in HLA-identical HSCT in the early phase, which was due to slower CD4^+^Tcons reconstitution. On the other hand, the recovery of Tregs after PTCy-haplo was earlier than that after HLA-identical HSCT, resulting in a significantly higher Tregs/Tcons ratio during the first 3 months after HSCT (101, 102). Although PTCy is a promising approach in terms of Treg reconstruction, further optimization of prophylaxis is required, including the optimal dose and duration of cyclophosphamide administration, the timing of calcineurin inhibitor initiation, and combining it with other immunosuppressant drugs.

## Effects of Other Immune Modulators on Treg Homeostasis

### Anti C-C Chemokine Receptor 4 Blockade

C-C chemokine receptor 4 (CCR4) is a chemokine receptor expressed in most patients with adult T-cell leukemia lymphoma (ATLL). Mogamulizumab (Mog) is a humanized anti-CCR4 immunoglobulin G1 monoclonal antibody that has an almost 50% efficacy in patients with relapsed or refractory ATLL (103, 104). CCR4 is also highly expressed on Tregs; therefore, pretransplant Mog may affect post-transplant Treg homeostasis and the incidence of GVHD. A multicenter, retrospective study demonstrated that pretransplant Mog was significantly associated with an increased risk of severe acute GVHD (105). Although a phase 1 study reported the half-life of Mog to be 15 to 18 days, retrospective data showed that the interval between the last administration of Mog to HSCT within 50 days was an independent prognostic factor for non-relapse mortality (105); therefore, the last treatment schedule of Mog is recommended to be ≥ 50 days prior to HSCT (106). In addition, the effect of Mog on Treg recovery after HSCT might be more profound than expected. We report a case of ATLL in which plasma exchange (PE) was conducted to eliminate residual Mog to treat severe GVHD (107). Although plasma Mog concentration was eliminated, it did not lead to the prompt elevation of Treg levels in peripheral blood, and the clinical responses of GVHD were limited to partial remission, suggesting that recovery of donor-derived Tregs in the acute phase after HSCT is multifactorial, and the single procedure of PE-based Mog depletion does not necessarily warrant the quick restoration of Treg homeostasis. As mentioned above, although Mog is a highly effective treatment for relapse and refractory ATLL, the increased risk of severe GVHD cannot be ignored. Avoiding the short interval between the last administration of Mog and HSCT and donors at high risk of GVHD should be considered to reduce the negative impact of Mog. Having said that, further clinical and basic research in this field is needed.

### α-Galactosylceramide

Although low-dose IL-2 therapy induced the selective expansion of Tregs in the chronic phase after HSCT, the specificity of the IL-2 effect may be shrunk in the acute phase after HSCT since activated effector T cells also express CD25 and may respond to the administered IL-2. In contrast, previous murine studies have demonstrated that invariant natural killer T (iNKT) stimulation mediated by α-galactosylceramide (α-GC) enables selective Treg expansion even in the very early phase following HSCT. iNKT cells are unique immunoregulatory T cell subsets that have limited TCR repertoires and recognize lipid antigens presented by CD1d on antigen-presenting cells (108). α-GC is a glycolipid originally purified from a marine sponge (109) and is also a ligand for iNKT cells in a CD1d-restricted manner. Previous studies have shown that α-GC stimulates different iNKT cell subsets depending on the method of injection (110). In the murine HSCT model, a single injection of αGC on day 0 after HSCT promoted Th2 polarization of donor T cells and the expansion of Tregs in a STAT6-dependent manner, which resulted in the reduced GVHD mortality (111, 112). Thereafter, a liposomal α-GC (lipo α-GC) was developed (113), and lipo α-GC was found to be safe and effective for acute GVHD prophylaxis in a murine model (114). Regarding the homeostasis of Tregs, a subsequent study revealed that host NKT cells induced an IL-4-dependent expansion of donor Tregs after HSCT (112). In addition, the adoptive transfer of donor iNKT cells ameliorated GVHD by expanding donor Tregs, in turn preserving the GVT effect (115). In addition to the acute GVHD model, our murine study using chronic GVHD models showed that α-GC treatment could ameliorate chronic GVHD symptoms through the early expansion of donor-derived Tregs followed by the suppression of follicular helper T cells and germinal center B cells (116). Based on the data of preclinical models, a phase 2 clinical study evaluated the efficacy of RGI-2001, a novel liposomal formulation of a synthetic derivative of α-GC in patients who underwent HSCT. A total of 29 patients received RGI-2001 on day 0 after HSCT, and 28% of recipients responded to RGI-2001 and increased the frequency and number of Tregs. These responders developed grade II to IV acute GVHD significantly less frequently than non-responders (117), suggesting that α-GC may prevent acute GVHD *via* Treg expansion in a clinical HSCT setting.

The combination of α-GC with other therapeutic modalities may be promising for further effective Treg modulation since the working mechanism of α-GC is unique and does not overlap with those of other therapies. Recently, we evaluated the effect of adding lipo α-GC after PTCy on GVHD and the GVT effect using the murine GVHD model (118). We demonstrated that a reduced dose of PTCy followed by adjuvant α-GC enhances the GVT effect without sacrificing GVHD suppression. Phenotypic analyses revealed that donor-derived B cells presented the ligand and induced preferential skewing to the NKT2 phenotype rather than the NKT1 phenotype, which was followed by the early recovery of all T cell subsets, especially Tregs. Our results propose the possibility of a novel strategy for optimizing PTCy-based transplantation.

### Anti-IL-2 Antibody

As the IL-2 receptors express on the activated effector T cells, the antibody for the IL-2 receptor has been investigated for GVHD prophylaxis and treatment. However, as IL-2 is an essential cytokine for Treg survival (45), the clinical effects of IL-2 receptor blockade appear not to be straightforward in patients after HSCT. Two IL-2 receptor antagonists, basiliximab and daclizumab, were added on the standard GVHD prophylaxis and explored the prophylactic effects against GVHD. These agents showed the preventive effect against acute GVHD, and basiliximab showed a superior prophylactic effect against chronic GVHD (119). However, the subsequent prospective randomized control study showed no additional prophylactic effect of daclizumab for acute GVHD, and rather it increased the risk of chronic GVHD and decreased the risk of disease recurrence (120). In the treatment setting, daclizumab has shown efficacy for a part of patients with steroid-refractory acute GVHD in the initial phase 2 trial (121), but the subsequent randomized control study showed a significantly worse survival rate in patients receiving corticosteroid with daclizumab than corticosteroid with placebo (122).

Denileukin diftitox is a genetically engineered protein composed of human IL-2 fused to diphtheria toxin and has cytotoxicity against activated T cells based on preferential binding to the high-affinity IL-2 receptor. A phase 1 study was conducted to evaluate the safety and efficacy of denileukin diftitox for 32 patients with SR-acute GVHD. Overall, 71% of evaluable patients achieved an overall response, including 33% of complete remission and 38% partial response (123). However, animal studies suggested that it could affect Treg homeostasis, and a case study reported that fatal hyperacute GVHD following denileukin diftitox treatment (124–126)

These inconsistent results may be attributed that anti-IL-2 antibody could affect the homeostasis of both Tregs and activated Tcons those expressing the IL-2 receptor. It suggests that anti-IL-2 antibody influence the patient’s immunity differently based on the balance between Tregs and activated Tcons in each patient basis, and the possible duality of the effect should be taken into account when these agents are used for patients after HSCT.

### Tyrosine Kinase Inhibitors

Standard GVHD prophylaxis in recipients with HLA-matched identical donor consists of the combination of calcineurin inhibitor (CNI) and short-term methotrexate. CNI reduced IL-2 transcription and activation of effector T cells and is a critical agent to regulate post-transplant immune reaction but have been shown to be disadvantageous in maintaining Tregs reconstitution because CNI inhibits the production of IL-2 from activated Tcons (127). Recently, novel immune modulating agents targeting Janus kinase (JAK), Bruton’s tyrosine kinase (BTK), or Interleukin-2 inducible tyrosine kinase (ITK) have been emerged.

Ruxolitinib (RUX) is a selective JAK1/2 inhibitor, and RUX reduced the GVHD histology score and prolonged survival in a murine GVHD model. RUX treatment suppressed the signals of inflammatory cytokines while sparing the IL-2–JAK3–STAT5 signal, which could lead to reduced CD4^+^IFN-γ^+^ cells and an increase of Tregs (128). In the human clinical setting, a phase 3 randomized trial compared the efficacy and safety of RUX with the investigator’s choice. The overall response was significantly higher in the RUX group, and RUX treatment significantly increased the median overall survival compared to control treatment (129).

Regarding BTK and ITK pathways, Ibrutinib, a BTK/ITK inhibitor, reduced the severity of chronic GVHD using two established chronic GVHD mice models (130). A phase 1b/2 study showed the safety and efficacy of ibrutinib treatment for patients with SR-cGVHD (131). While the significance of BTK inhibition on Treg homeostasis remains unknown, some studies showed that ITK inhibition might increase Tregs in murine models (132). Mammadli et al. reported that ITK-deficient donor T cells significantly reduced inflammatory cytokines leading to less GVHD intensity in the mice model (133). We recently demonstrated that pharmacological inhibition of ITK on donor T cells could ameliorate acute GVHD without sacrificing the GVT effect (134). In the study, we showed that ex-vivo graft manipulation with ITK inhibitor modulated donor CD4^+^ T cell differentiation towards Th1, Th2, and Th17 with sparing Tregs, resulting in the prolonged overall survival after HSCT.

### Adoptive Transfer of Tregs

In addition to the pharmacological *in vivo* modulation of Treg homeostasis described above, the GVHD-preventive effect of adoptive transferred donor-type Tregs has been evaluated in murine models and clinical studies. The adoptive transfer of Tregs enables to increase in the Treg pool at least transiently, and it may provide merit especially in patients having insufficient thymic recovery. The initial experimental study demonstrated that the adoptive transfer of Tregs to the graft suppressed the expansion of alloreactive donor T cells without impairing the GVT effect (18). In a human acute GVHD prophylaxis setting, a first in human clinical trial evaluated ex-vivo expanded umbilical cord blood (UCB)-derived Tregs for patients receiving nonmyeloablative double UCB transplantation (135). Tregs were isolated and cultured with anti-CD3/anti-CD28 monoclonal antibody-coated beads supported by IL-2. A total of 1-30 x 10^5^/kg UCB-derived Tregs infused on day+1 for all participants and day+15 for an additional cohort. GVHD prophylaxis consisted of cyclosporine and mycophenolate mofetil (MMF), but later changed to sirolimus and MMF due to potential interference with Treg function and survival by cyclosporine. Grade II to IV acute GVHD and the incidence of chronic GVHD were lower than the historical control. The subsequent study from the same group demonstrated the safety and efficacy of UCB-derived Treg expanded with K562 cells. This novel approach enabled the expansion of Tregs to up to 30 times higher than the previous method. In this study, the rate of grade II to IV acute GVHD was lower than the control without increasing the infection and relapse rates (136). The Perugia group infused Tregs followed by stem cell and Tcons infusion without immunosuppression therapy after transplantation. They conducted a phase 2 study evaluating the effect of isolated donor Tregs (1 x 10^6^/kg) on day-4, followed by a purified CD34^+^ and Tcons (1 x 10^6^/kg) on day0 without posttransplant immunosuppression for patients receiving HLA-haploidentical transplantation (137). In this setting, purified Tregs and Tcons were harvested from the same donor as the stem cell harvest and not cultured ex-vivo. The incidence of grade II to IV acute GVHD was 15%, and this rate of acute GVHD was similar to the historical controls. Surprisingly, although the high-risk patients’ background, the relapse rate was significantly lower than the historical control group, suggesting that adoptive transfer of Tregs followed by Tcons and CD34^+^ stem cells as acute GVHD prophylaxis strategy suppress GVHD without abrogating the GVT effect in a human setting. Recently, a subsequent study involving fifty HLA-haploidentical transplant recipients with Treg adoptive transfer showed excellent results (138). Fifteen patients developed grade II to IV acute GVHD, including 12 grade III to IV, and all recipients were treated with corticosteroids. Only 5 patients were refractory to first-line steroid therapy, and 3 of them recovered after the second-line treatment. Of note, only 2 patients relapsed, and the moderate/severe chronic GVHD/relapse-free survival was 75%.

On the contrary, the data of adoptive transfer of Tregs in the treatment setting of active ongoing acute GVHD treatment was limited. Two case series described the use of ex-vivo expanded Treg infusion for the treatment of acute GVHD and chronic GVHD (139, 140). These studies demonstrated the feasibility of the ex-vivo Treg expansion and infusion, but the effect of this approach should be evaluated by larger clinical trials.

To maintain transferred Tregs *in vivo*, low-dose IL-2 administration after adoptive transfer of Tregs is theoretically attractive. In a phase 1 study, 24 SR-cGVHD patients received freshly isolated Tregs from the original stem cell donor followed by IL-2 (1 x 10^6^ U/m^2^/day) treatment for 8 weeks. The response rate was 33%, and TCRβ diversity in Tregs is normalized with therapy (141). Larger clinical trials are warranted to confirm the efficacy of this combination therapy in patients with SR-cGVHD.

## Emerging Problems and Future Perspectives

Ever since the discovery, Tregs have been the focus of many studies owing to the efficient immunosuppressive function. In the allogeneic HSCT, as Tregs have a central role in the regulation of post-transplant immunity, the dynamics of Tregs have been extensively studied in murine models and clinical samples. As described in this review, Tregs originally have different characteristics in homeostasis from conventional T cell subsets, and understanding the unique homeostasis makes it possible to give Tregs selective effects *in vivo*. However, there remain many unresolved questions about Treg homeostasis in patients.

First, one of the critical problems is the localization of Tregs in a human clinical setting. Previous studies suggest that the phenotype and function of Tregs are different depending on the localization, such as lymph nodes, target tissues, and tumor micro-environments. For example, in patients with gastric cancer, Treg phenotype is different between tumor infiltrating Tregs and peripheral circulating Tregs (68). Most of human GVHD clinical studies depended on the circulating Tregs, hence, an evaluation of Treg localization and the function in each specific site would be helpful for our further understanding of Treg homeostasis in the context of GVHD.

Second, the impact of Tregs on the GVT effect has not been well characterized in human clinical transplant (142). To further elucidate the Treg effect on the GVT, further research is needed to develop clinically-relevant murine models to study anti-tumor immunity after allogeneic HSCT.

Third, the effect of novel immunotherapies on the Tregs has not been well studied. Recent developments of immunotherapies such as chimeric antigen receptor T cell (CAR-T), bispecific T-cell engagers (BiTE), and ICIs have provided significant progress for treatment. Despite improvement in the response rate, patients with refractory diseases still often require post-remission therapy with HSCT. The effects of a previous history of CAR-T or BiTE on subsequent allogeneic HSCT are largely unknown. In particular, it is important to study the effect of pretransplant novel immunotherapies on donor-derived immune cell recovery, including Tregs, after allogeneic HSCT.

Advances in the understanding of Treg homeostasis in patients after HSCT may contribute to the treatment for autoimmune diseases and solid cancers. In solid cancers, Tregs negatively influence tumor control; therefore, contrary to GVHD, reducing Tregs and activating effector T cells may promote the anti-tumor effect. Based on our previous reports regarding the role of PD-1 on Tregs after low-dose IL-2 and HSCT (76, 86), the combination treatment IL-2 and PD-1 blockade may activate effector T cell and promote Treg apoptosis in the inflammatory environment, like a tumor microenvironment.

Novel experimental methodologies may enable us to overcome the remaining issues shown here. In fact, the single-cell technique made detailed characterization of cells and their microenvironment, and to answer questions that could not be addressed by the conventional technique (143). In the context of acute GVHD, scRNA-seq has identified a novel regulator of T cell alloimmunity (144). Previous research has investigated the role of long noncoding RNA after HSCT using scRNA-seq and identified *Linc00402* as a regulator of allogeneic T cell function. In addition, the TCR sequence method provides information on the TCR diversity and clonality of these populations. Since each T cell possesses its own unique TCR, TCR-seq allows for sensitive tracking of T cells at the clonal level (145). These novel approaches may solve the limitations of previous studies by conventional approach and provide a bridge to new findings in Treg research.

## Author Contributions

SI wrote the paper. K-iM designed and edited the paper. All authors contributed to the article and approved the submitted version.

## Funding

This work was supported by JSPS KAKENHI grant 26461449.

## Conflict of Interest

The authors declare that the research was conducted in the absence of any commercial or financial relationships that could be construed as a potential conflict of interest.

The reviewer JK declared a shared affiliation with one of the authors SI, with no other collaboration, at the time of review to the handling editor.

## Publisher’s Note

All claims expressed in this article are solely those of the authors and do not necessarily represent those of their affiliated organizations, or those of the publisher, the editors and the reviewers. Any product that may be evaluated in this article, or claim that may be made by its manufacturer, is not guaranteed or endorsed by the publisher.
